# Lessons Learned from Targeting IGF-I Receptor in Thyroid-Associated Ophthalmopathy

**DOI:** 10.3390/cells10020383

**Published:** 2021-02-12

**Authors:** Joseph A.M.J.L. Janssen, Terry J. Smith

**Affiliations:** 1 Erasmus Medical Center, Department of Internal Medicine, Dr. Molewaterplein 40, 3015 GD Rotterdam, The Netherlands; 2 Kellogg Eye Center, Department of Ophthalmology and Visual Sciences, University of Michigan Medical School, Ann Arbor, MI 48105, USA; terrysmi@med.umich.edu; 3 Division of Metabolism, Department of Internal Medicine, Endocrinology, and Diabetes, University of Michigan Medical School, Ann Arbor, MI 48105, USA

**Keywords:** Graves’ disease, thyroid-associated ophthalmopathy, proptosis, IGF-IR, IGF-IR inhibitors, T cells, B cells, TSHR, autoimmunity

## Abstract

Complex immunological mechanisms underlie the pathogenesis of thyroid-associated ophthalmopathy (TAO). Historical models of Graves’ disease and TAO have focused almost entirely on autoimmune reactivity directed against the thyrotropin receptor (TSHR). The insulin-like growth factor-I receptor (IGF-IR) has been proposed as a second participating antigen in TAO by virtue of its interactions with IGFs and anti-IGF-IR antibodies generated in Graves’ disease. Furthermore, the IGF-IR forms with TSHR a physical and functional complex which is involved in signaling downstream from both receptors. Inhibition of IGF-IR activity results in attenuation of signaling initiated at either receptor. Based on the aggregate of findings implicating IGF-IR in TAO, the receptor has become an attractive therapeutic target. Recently, teprotumumab, a human monoclonal antibody IGF-IR inhibitor was evaluated in two clinical trials of patients with moderate to severe, active TAO. Those studies revealed that teprotumumab was safe and highly effective in reducing disease activity and severity. Targeting IGF-IR with specific biologic agents may result in a paradigm shift in the therapy of TAO.

## 1. Biology of Insulin-Like Growth Factor (IGF) Family and Their Receptors and Associated Proteins

The IGF/insulin family consists of three activating ligands (IGF-I, IGF-II, and insulin), four receptors IGF-IR and IGF-IIR (also known as the mannose-6 phosphate receptor), and insulin receptor A (IR-A), and IR-B, six IGF-binding proteins (IGFBP1-6) and nine IGFBP-related proteins (IGFBP-rPs) [1]. Research in the last 50 years has uncovered the molecular structures of these molecules. IGF-I and IGF-II exhibit substantial structural homology; both consist of A-domains and B-domains, which are homologous to those respective regions of insulin [2]. Amino acids comprising IGF-I and IGF-II possess a 50% identity to proinsulin, the precursor of insulin [2]. In contrast to insulin, the C-domains of the mature IGFs are retained [3]. Compared to proinsulin, both IGF-I and IGF-II contain an additional D-domain extending from the C-terminal end of the A-chain [3]. IGF-I forms a single chain of 70 amino acids with a calculated molecular weight of 7649 Daltons [2]. IGF-II contains 67 amino acids with and has a calculated molecular weight of 7500 Daltons [4]. Both IGF-I and IGF-II contain three intra-molecular disulfide bridges. IGF-II is one of the most abundant growth factors of the body and is the most abundant peptide with insulin-like activity [5].

IGF-IR, IR-A, and IR-B belong to the family of ligand activated receptor kinases, while IGF-IIR lacks receptor kinase activity [6]. IGF-IR and IRs share both structural and functional homology [6]. Depending on specific regions, IGF-IR and IRs have sequence similarities of 41–84% [7]. The structural similarities between IGF-IR and IRs result in substantial ligand promiscuity [8]. IGF-IR binds IGF-I and IGF-II with a Kd ~10^−9^–10^−10^ M but its affinity for insulin is 100-fold lower [9]. In contrast, insulin binding to IRs is very high-affinity (Kd ~ 10^−10^ M), 10-fold lower for IGF-II and 50–100 fold lower for IGF-I [9]. In this respect the IR-A and IR-B differ: insulin and IGF-II have a higher affinity for IR-A than for IR-B [10]. Both IGFs primarily activate IGF-IR, while insulin and IGF-II primarily activate the IR-A and insulin primarily activates IR-B. Activation of both IGF-IR and IR-A results in cell growth, proliferation, and enhanced cell survival. In contrast, IR-B activation induces metabolic processes [11]. Differences in IGF-IR and IR-B activities observed in vitro appear to result from differences in the relative expression levels of the two proteins [6].

**Figure 1:**
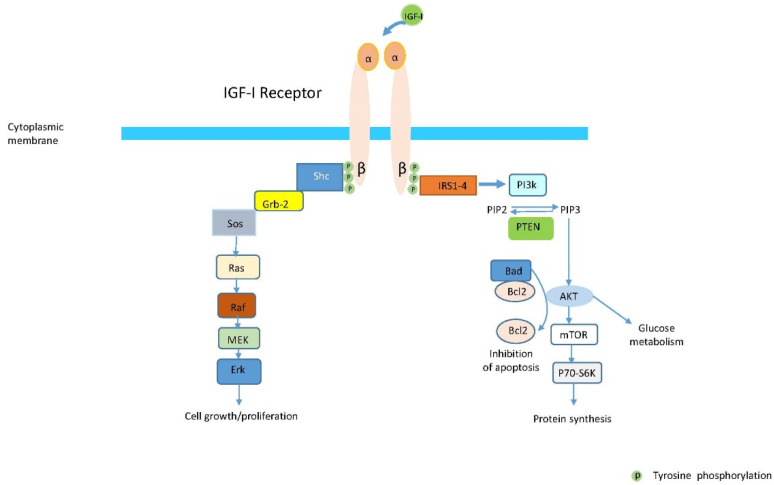
Binding of IGF-I to the extra-cellular alpha subunit induces a conformational change of the IGF-IR, which enables autophosphorylation of the intrinsic tyrosine kinases domains of the beta subunits of the IGF-IR. In the classical model of signaling, this was considered the first step in the intracellular signaling cascade of post-receptor events. This further results in downstream stimulation of either phosphatidylinositol 3 kinase (PI3K/AKT) and mTOR or MEK (ERK1 and ERK2) proteins leading the activation of different genes and the initiation of different cellular processes. This involves the participation of many cytoplasmic proteins such as IRS-1, PI3K, PTEN. AKT, mTOR, and SK6 in one signaling branch and Shc, Grb2, Sos, Ras, Raf, MEK and ERK in a second signaling branch.

Unlike most RTKs, IGF-IR and IRs are covalently linked dimers comprising two extracellular α-subunits and two transmembrane β-subunits joined by disulfide bridges. They are both plasma membrane-spanning and remain dimeric regardless of bound ligand status [7]. Ligand binding to the extra-cellular α subunit results in a conformational change enabling endogenous tyrosine kinase autophosphorylation occurring in the β subunits [12]. The classical signaling model envisaged this as representing the first step in downstream signaling through MAPK/Ras-Raf-ERK, PI3K/AKT and FRAP/mTOR [13,14] (Figure 1). Evidence now suggests tyrosine kinase-independent functions are also associated with IGF-IR [15]. In addition, IGF-IR may initiate post-receptor pathway signaling in an unligated state through an unidentified mechanism(s) [16]. Boucher et al. demonstrated that cells without either IGF-IR or IR express lower levels of multiple imprinted genes and microRNAs [16].

More recently, IGF-IR (intact holo-IGF-IR or as a free β-subunit) has been shown to translocate into the nucleus after ligand binding the α-subunit [17] while another study demonstrated α-subunit nuclear translocation in fibroblasts from patients with Graves’ disease [18]. Several studies have suggested that nuclear IGF-IR can bind the IGF-IR gene promoter region and in so doing, induce its own expression [15,17,19]. Moreover, upon nuclear localization IGF-IR may stimulate TCF-mediated β-catenin transcriptional activity, which is a key nuclear effector of canonical Wnt signaling [17,20]. Both ligand binding and tyrosine kinase activity are considered to be critical for the nuclear localization of IGF-IR [21,22]. However, Jamwal et al. have suggested that cytoplasmic and nuclear activities of IGF-IR should be considered two independent functions [17].

**Figure 2:**
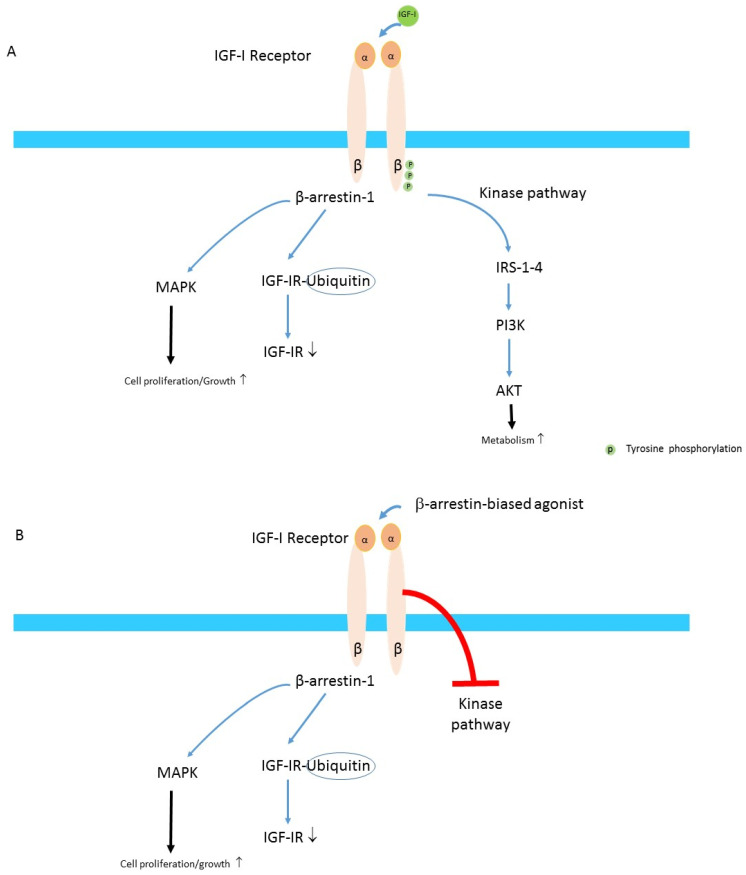
(**A**) Balanced agonism. Binding of IGF-I to the IGF-IR induces stimulates both the β-arrestin-1 pathway that leads to MAPK activation and proteasome degradation of the IGF-IR through an ubiquitin-mediated mechanism resulting in a loss of the number of IGF-IRs at cellular membrane. IGF-I binding to the IGF-IR also induces phosphorylation of tyrosine residues and this stimulates the kinase pathway which finally activates AKT. (**B**) β-arrestin-1-biased agonism. Binding of a biased agonist preferentially increases β-arrestin-1 signaling and this simultaneously inhibits the IGF-IR kinase pathway. Thus, β-arrestin-1-biased agonism results in down-regulation of the IGF-IR and inhibition of AKT signaling, but increases MAPK-mediated cell proliferation and growth (Modified from Salisbury & Tomblin. Insulin/Insulin-like growth factors in cancer: new roles for the aryl hydrocarbon receptor, tumor resistance mechanisms, and new blocking strategies [26].

In addition to the aforementioned signaling pathway use, IGF-IR can also activate components of GPCR pathways and thus might be considered to be functional tyrosine kinase/GPCR hybrids, integrating kinase signaling with IGF-IR-mediated GPCR features [15,23,24]. IGF-IR may exhibit homologous and heterologous desensitization and biased agonist-like behavior such as that historically considered typical for classical GPCRs [15]. Down-regulation and degradation of the IGF-IR after stimulation by a biased agonist may not only inhibit the “classical” RTK pathways, but paradoxically might stimulate β-arrestin-1-dependent MAPK-pathway activation [25] (Figure 2A,B).

In hybrids α-β IGF-IR subunit are linked by disulfide bonds to α-β IR. Both IR-A/IGF-IR (Hybrid A) as well IR-B/IGF-IR (Hybrid B) receptors can be formed [27]. The biological functions of these hybrids remain uncertain without examining cells not expressing holo-IGF-IR or holo-IR [28]. Nevertheless, it has been suggested that IR/IGF-IR hybrids act in a manner similar to that of IGF-IR. Hybrid receptors respond more predictably to IGF-I than to insulin under physiological conditions [28,29]. They may account, at least in part, for the overlapping yet distinct actions of the two ligands. Holo-IGF-IR and hybrid receptors promote both cell proliferation and glucose uptake, contrasting with holo-IR which only enhances glucose uptake. In contrast, holo-IGF-IR can mediate both mitogenic and anti-apoptotic effects [28].

IGF-IR can also form hybrids with RTKs not belonging to the insulin-IGF family [15]. For instance, IGF-IR and EGFR can pair, enabling the former to activate signaling pathways downstream from EGFR [30]. On the other hand, inhibition of one protein component of a hybrid can shift signaling toward its counterpart [31]. IGF-IR can further transactivate signaling pathways of GPCRs via chemokine receptors, which in turn are capable of EGFR activation [32]. Signaling initiated through IGF-IR may also interact with that downstream of TSHR, a classical GPCR [33] and the two may overlap [34]. Tsui et al demonstrated that IGF-IR and TSHR form physical and functional complexes as plasma membrane-spanning receptors [34]. Co-immunoprecipitation/pull-down techniques and confocal microscopy of orbital fibroblasts, thyrocytes and in situ in orbital tissue demonstrated these complexes [34]. Since Tsui et al. also observed that a specific monoclonal antibody directed against and inhibitory to IGF-IR could block rhTSH and GD-IgG mediated ERK phosphorylation in primary thyrocytes, they suggested that IGF-IR might attenuate downstream signaling initiated by TSHR [34]. Those concepts are further supported by observations that the IGF-IR activity is required for thyrocyte growth promoted by TSH/IGF-I in vitro [35] and that TSH-stimulated goiter is completely inhibited in mice by thyrocyte-selective IGF-IR knockout [35]. More recent findings from Krieger et al. demonstrated that simultaneous activation of the IGF-IR and TSHR resulted in synergistic activation of ERK1 and ERK2 in multiple cell types [36].

Extracellular IGF is predominantly bound to IGFBPs [37]. These IGFBPs share a conserved structural organization; a C-terminal and N-terminal domain are uniformly present. These are connected by a variable central linker domain [38]. IGFBPs bind IGF-I and IGF-II with equal or greater avidities than those of IGF-IR [39]. Despite their significant structural homologies, the six IGFBPs possess distinct functions. While circulating, they serve as IGF carriers, modulating circulating IGF disposal while regulating ligand/IGF-IR interactions [39]. Circulating IGFBP-3 and IGFBP-5 can form ternary complexes with IGF and the acid labile subunit [39]. Thus, IGFBPs act as circulating “IGF reservoirs”, during acute need such as during hyperglycemia while modulating IGF-I actions [38]. Changing circulating IGFBP concentrations allows modification of “free” (i.e., biologically available) IGF fractions [40]. IGFBPs are widely expressed in most tissues and function as autocrine/paracrine regulators of IGF activity [41,42]. Multiple hormones such as glucocorticoids, androgens, estrogens and insulin regulate IGFBP synthesis and bioavailability [43]. Thus, in addition to their endocrine roles as carrier proteins, IGFBPs can modulate IGF activity in peripheral tissues [39]. Moreover, IGFBPs can act through IGF-independent mechanisms [42]. It has become increasingly clear that they influence essential cellular processes through both IGF-dependent and -independent actions [42].

Several IGFBP proteases and their respective circulating inhibitors govern the abundance of IGFBPs, allowing customization of IGF activities tailored to specific physiological circumstances [41]. In states of health, low-level circulating IGFBP protease activity can be detected, the consequence of protease inhibitors. The balance between these proteases and inhibitors regulates IGF-IGFBP complex intactness [41]. As consequence, circulating free IGF concentrations are maintained at low levels; however, free IGF concentrations can increase considerably under special circumstances, such as pregnancy. Variations in inhibitor levels rather than IGFBP protease concentrations seem to underlie changes in IGFBP-IGF complex levels [44]. In contrast, influence of these IGFBP protease inhibitors on IGFBP/protease balance appears less important in interstitial fluids [41], rendering protease activity unopposed [41]. Consistent with this possibility, IGF-IR stimulating activity, as assessed by IGF-IR kinase receptor activation assay is increased in interstitial fluids compared to serum [45].

## 2. Role of IGFs and IGF-IR in Regulating Growth and Development

Growth hormone (GH) can directly stimulate tissue growth in many organs, such as muscle, bone, and cartilage; however, many actions by GH are mediated indirectly through those of IGF-I. GH is one of several factors regulating circulating IGF-I levels, mainly produced in liver. Other hormones such as thyroxine, cortisol, and sex steroids interact with GH by regulating hepatic IGF-I synthesis [46]. The immune system also plays an important role in IGF-I synthesis in liver [47]. Diet also influences IGF-I levels [47,48]. Hepatic IGF-I production accounts for about 80% of total serum IGF-I in experimental animals [49]. The remainder is synthesized locally in many if not all other tissues [42]. In the original somatomedin hypothesis, circulating IGF-I was considered the key component in regulating growth [50]. In that concept, GH secreted by the pituitary gland regulated somatic growth solely by controlling hepatic IGF-I (AKA somatomedin C) production [50]. Several decades ago, the original hypothesis was challenged by several important observations in liver-specific IGF-I gene knock-out (KO) mice (Liver IGF-I^−/−)^ [49,51]. Those IGF-I^−/−^ animals grew normally compared to wild-type littermates (body weight, body length, and femoral length), despite circulating IGF-I level reductions to less than 25% of those in intact animals [49]. This model provided direct evidence for the importance of autocrine/paracrine IGF-I in postnatal growth [49]. Currently, IGFs produced in extra-hepatic tissues is considered to substantially contribute to somatic growth [46,52]. In addition, tissue-specific factors other than GH may play a role and stimulate or inhibit IGF-I production in a variety of tissues [53].

Stimulation of IGF-IR by IGF-I plays a significant role in stem cell proliferation and differentiation [17,54,55,56]. IGF-I appears important for the maintenance of tissue resident adult stem cells [57]. The growth-promoting effects of IGF-I via actions through IGF-IR include stimulation of DNA synthesis, cell proliferation, differentiation, and migration of many cell types [1,58]. It inhibits apoptosis (programmed cell death) and repair (maintenance) of many tissues [59,60]. Other anabolic actions of IGF-I include both stimulating glucose- and amino acid uptake as well as RNA and protein synthesis [3]. In addition, IGFs may act as permissive factors to augment signals of other (hormonal) factors [58]. For example, while TSH represents the principal regulator of thyroid hormone biosynthesis and thyroid growth, IGF-I synergistically augments its functions in promoting thyrocyte growth in vitro [35]. Patients with the Laron syndrome (also known as congenital GH insensitivity) fail to respond to GH and thus fail to generate IGF-I, developing dwarfism [61]. Diminished head circumference and underdeveloped facial structures characterize these patients. These individuals represent a unique model for the roles of IGF-I in development of the human orbit [61] and the eye [62]. They manifest reduced axial and anterior chamber lengths of the globe, defects which can be reversed with IGF-I therapy [62]. Growth of individual organs might be uncoupled from that of overall body growth, a divergence potentially involving IGF-IR [63].

## 3. Role of the IGF-I Pathway in Regulating Immune Function

IGF-I, IGF-IR, IGFBPs, and related molecules exert important influence on host defense and immune responses. Many components of this pathway are expressed by professional immune cells [64]. Moreover, IGF-I regulates several aspects of immune activities in myeloid, lymphoid and hematopoietic cell types through endocrine, autocrine and paracrine actions [65]. Both innate and adaptive immune systems are affected by IGF-I, insulin and IGFBPs [66]. IGF-I can restore both B and T cells following lethal irradiation and subsequent bone marrow grafting [67,68]. It enhances mature B cells and plasmocyte proliferation as well as antibody responses [69]. IGF-IR expression is widespread in peripheral mononuclear cells [70,71,72], including CD4^+^ and CD8^+^ T cells, B cells, monocytes, natural killer cells and thymocytes. These cells display high level IGF-IR [73,74,75,76,77,78,79,80,81,82], although the binding affinities and expression levels vary [70,74]. IGF-I increases responsiveness of T and B cells isolated from spleen and lymph nodes to mitogen and antigen stimulation [65]. Human macrophages and granulocytes also express surface IGF-IR [83,84] and IGF-I inhibits apoptosis while enhancing cytokine and chemokine expression, including tumor necrosis factor α (TNF-α), IL-8 [85], and IL-2 [86]. In contrast, IGF-I can also induce anti-inflammatory cytokines such as IL-10 [87].

## 4. IGF-IR and TAO

**Figure 3:**
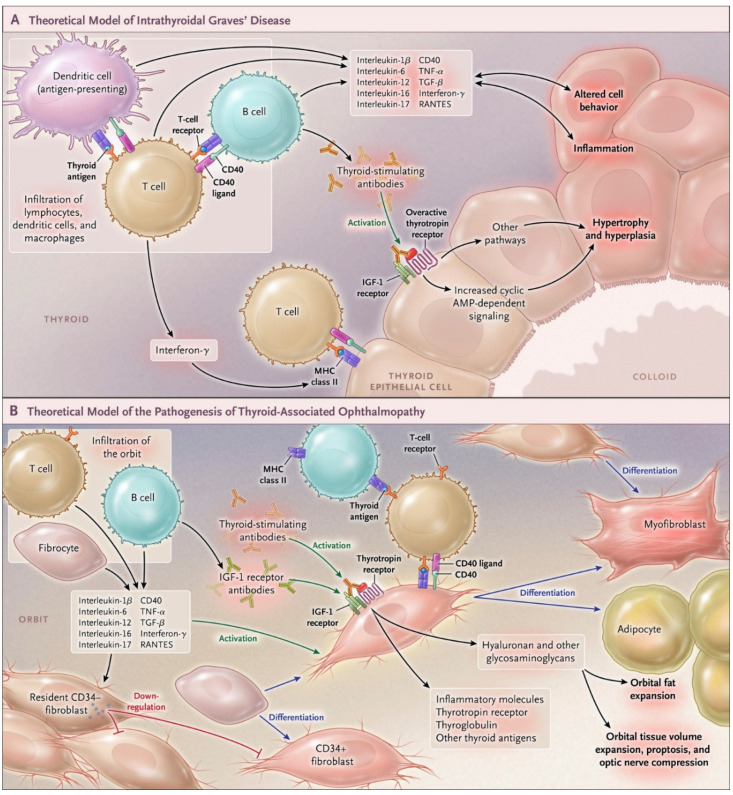
Involvement of IGF-I receptor in the pathogenesis of Graves’ disease and thyroid and thyroid-associated ophthalmopathy. (**A**) Theoretical model of how Graves’ disease occurs in the thyroid gland. Thyroid-stimulating immunoglobulins over-stimulate thyroid hormone production mediated through the thyrotropin receptor. Infiltrating B and T cells and antigen-presenting cells produce multiple cytokines. These include interleukins 1β, 6, and 12; interferon-γ; tumor necrosis factor α; and CD40 ligand. These result in inflammation. (**B**) Theoretical model of thyroid-associated ophthalmopathy. Orbit-infiltrating B and T cells and CD34^+^ fibrocytes interact, resulting in inflammation and tissue remodeling. Fibrocytes differentiate into orbital fibroblasts and further into myofibroblasts or adipocytes. Both residential and infiltrating cells can produce cytokines, including interleukins 1β, 6, 8, and 16; tumor necrosis factor α (TNF-α); RANTES (regulated on activation, normal T-cell expressed and secreted); and CD40 ligand. Fibrocyte-derivative CD34^+^ fibroblasts express low-level thyrotropin receptor, thyroglobulin, and thyroperoxidase. Pathogenic IgGs activate the thyrotropin– insulin-like growth factor 1 (IGF-1) receptor complex, resulting in production of inflammatory molecules and glycosaminoglycans. From N. Engl. J. Med, Smith T.J. and Hegedus L., Graves’ Disease, 375; 1552–1565. Reprinted with permission from ref. [89]. Copyright 2016 Massachusetts Medical Society.

With regard to GD and TAO, the centrality of IGF-IR [88] is captured in the image shown in Figure 3A,B [89]. Initial connections between the IGF-I pathway and TAO pathogenesis dates to the observations that IgGs from patients with the disease could displace radiolabeled IGF-I from binding sites on orbital fibroblasts from these patients [90]. A larger fraction of T cells express IGF-IR in patients compared to healthy donors [91]. In addition, a large component of T-cell expansion localizes to those with the CD45RO^+^ IGF-IR^+^ phenotype, apparently the consequence of resistance to apoptosis. Specifically, T cells in this subset resist Fas-mediated apoptosis as well as exhibiting increased proliferative rates. Circulating IGF-IR^+^ B cells are disproportionally represented in GD, produce antibodies and enhanced survival in response to IGF-I [92]. Therapies targeting both T and B cells are currently being investigated [93].

Besides immune cells, fibroblasts from the TAO orbit (GD-OF) also express higher IGF-IR levels than do those from healthy donors [34,94]. Several findings suggest the importance of IGF-IR displayed on these fibroblasts in the pathogenesis of TAO. IgGs from patients (GD-IgG) bind IGF-IR and initiate signaling in GD-OF, responses that are absent in those from healthy individuals [94,95,96]. In addition, signaling initiated through TSHR is dependent on IGF-IR for downstream signaling to MAPK p42/44 ERK [34]. IGF-IR and TSHR form a physical and functional complex in GD-OF, in situ in TAO orbital fat, and in primary thyroid epithelial cells [34]. IGF-I levels have been found elevated in TAO orbital fat and extraocular muscle [97,98]. It would appear that the IGF-I pathway plays potentially important roles in the pathogenesis of TAO.

## 5. Teprotumumab Has Proven Effective and Safe in Moderate to Severe, Active TAO in Two Clinical Trials

### 5.1. Phase 2 Trial

**Figure 4:**
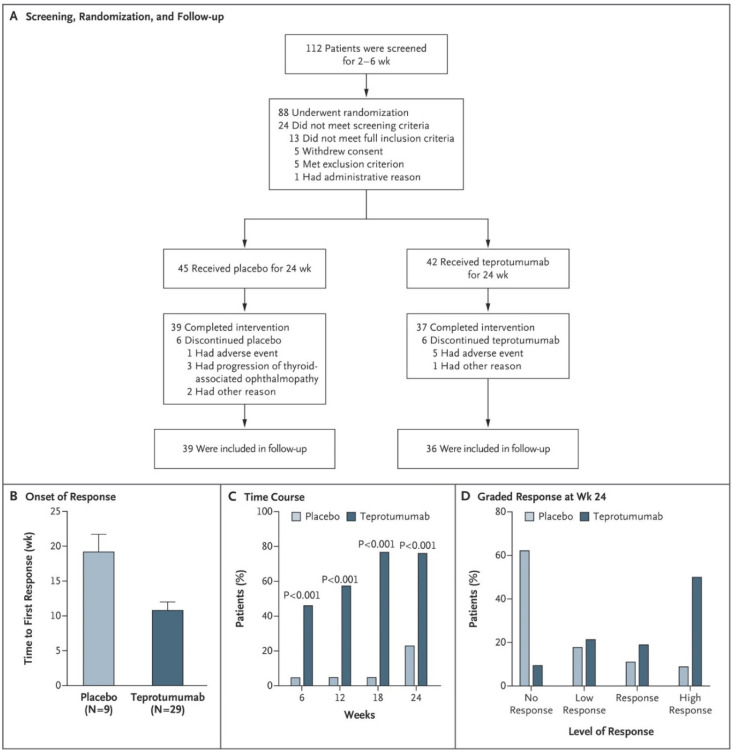
Screening, randomization, response, and follow-up in a phase 2 clinical trial of teprotumumab. (**A**) Patients were screened, and randomized to receive either active drug or placebo for the 24-week treatment phase. They were then followed for 1-year. (**B**) Analysis to first response. (**C**) Time course of patients meeting primary response criteria. (**D**) Responses were graded at week 24. High response indicates ≥ 3 mm proptosis and ≥ 3 point improvement in clinical activity score (CAS) using a seven-point scale. From Smith TJ, Kahaly GJ, Ezra DG, Fleming JC, Dailey RA, Tang RA, Harris GJ, Antonelli A, Salvi M, Goldberg RA, Gigantelli JW, Couch SM, Shriver EM, Hayek BR, Hink EM, Woodward RM, Gabriel K, Magni G, Douglas RS. Teprotumumab for thyroid-associated ophthalmopathy. N Engl J Med. 2017;376(18):1748–1761. Reprinted with permission from ref. [99]. Copyright Massachusetts Medical Society.

An array of studies, conducted almost entirely in vitro, established a plausible use of IGF-IR inhibitors as therapy for TAO. Those findings culminated in the organization of a clinical trial of the drug, teprotumumab, also known as RV001 or R1507, beginning in 2010. The phase 2 study was sponsored by River Vision after teprotumumab failed to demonstrate requisite effectiveness as a cancer treatment. The study was placebo-controlled, multi-centered, and double-masked. Eighty-eight eligible patients with active (clinical activity score (CAS) ≥4 on a 7-point scale), moderate to severe TAO were randomized to receive infusions every 3 weeks for a total of eight doses over a 24-week treatment phase of either active drug or placebo [99]. A total of 15 sites enrolled clinically euthyroid patients between 18 and 75 years of age who had begun to manifest TAO within 9 months of trial baseline. They had no history of remedial ocular surgeries for TAO or treatment with immune-suppressive drugs or >1 g of prednisone equivalent (with a required 6-week washout period from all systemic steroids). Enrolment began 2 July 2013 and was completed 23 September 2015. The intention to treat (ITT) cohort (87 patients) were assigned randomly to either the placebo group (*n* = 45) or those receiving active drug (*n* = 42). The primary outcome, an aggregate of a ≥2-point improvement of CAS (using a 7-point scale) and a reduction of ≥2 mm in proptosis in the study eye without a similar worsening in the fellow eye at week 24. Secondary endpoints included improved CAS ≥2 points and proptosis reduction of ≥2 mm from baseline, measured as independent variables, improved diplopia and improvement of quality of life using a validated questionnaire (GO-QoL) [100]. Any subject entered into the ITT cohort and failing for any reason to receive any of the protocol-mandated doses or to undergo assessment at week 24 was considered a treatment failure. Adverse events were also assessed for occurrence and severity. Processes of screening, randomization and follow-up are included in Figure 4A–D. Patient characteristic of the two treatment arms at baseline were similar with regard to demographics, duration of disease, CAS, thyroid hormone and thyroid-stimulating immunoglobulin levels. Moreover, baseline proptosis, clinical activity, and GO-QOL scores were similar; however, the frequency of graded diplopia prior to therapeutic intervention differed in the two groups. Efforts to stratify patients with regard to cigarette smoking were unsuccessful and an imbalance was detected; fewer smokers were included in the cohort receiving active drug.

**Figure 5:**
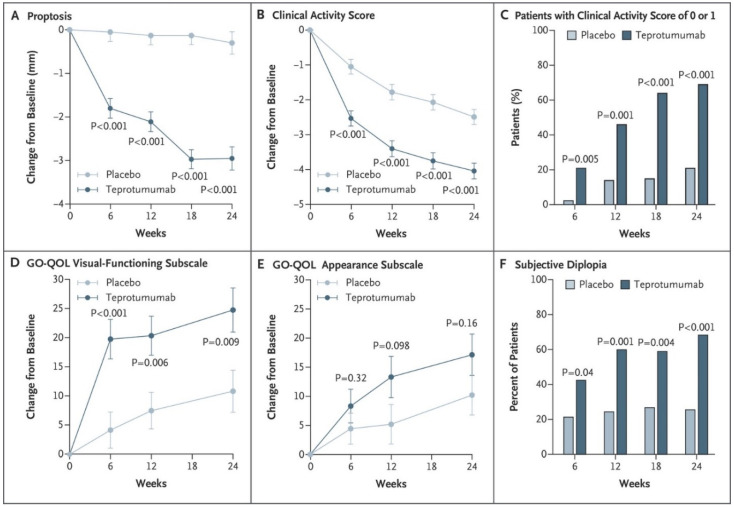
Secondary end points in phase 2 clinical trial of teprotumumab. (**A**) Changes in proptosis over time from baseline. (**B**) Changes in CAS over time from baseline. (**C**) Post hoc analysis showing fraction of patients achieving 0–1 CAS as a function of time. (**C**) Change in QOL visual functioning subscale (GO-QOL). (**D**) GO-QOL visual functioning subscale. (**E**) Change in GO-QOL appearance subscale. (**F**) Diplopia responses. From Smith TJ, Kahaly GJ, Ezra DG, Fleming JC, Dailey RA, Tang RA, Harris GJ, Antonelli A, Salvi M, Goldberg RA, Gigantelli JW, Couch SM, Shriver EM, Hayek BR, Hink EM, Woodward RM, Gabriel K, Magni G, Douglas RS. Teprotumumab for thyroid-associated ophthalmopathy. N Engl J Med. 2017;376(18):1748–1761. Reprinted with permission from ref. [99]. Copyright Massachusetts Medical Society.

Results from the study revealed that a similar number of patients in the two treatment groups completed the 24-week intervention phase (87% versus 88%). 29/42 (69%) patients in the ITT group who received teprotumumab achieved the primary response at 24 weeks while 9/45 (20%) of patients in the placebo arm responded (*p* < 0.001). Those receiving active drug exhibited a shorted time to response. Nearly half of the patients in the teprotumumab group responded at the 6-week assessment (*p* < 0.001 versus placebo). The differences in the two groups continued to widened over the course of the 24-week treatment so that at the final assessment, 49% more patients receiving the active drug responded (*p* < 0.001). Considerably more patients achieved a high response at week 24 (defined as ≥3 point improvement in CAS combined with ≥3 mm proptosis reduction in the study eye). The achievement of secondary outcomes was congruent with that of the primary response, being similarly skewed to those receiving active drug (Figure 5A–F). Both proptosis and CAS improved from baseline assessments at week 24. Improvement in the GO-QOL score was limited to the vision subscale; patients receiving teprotumumab failed to exhibit a significant difference from the placebo group with regard to appearance. Diplopia was improved significantly more in those receiving teprotumumab than those in the placebo group. The FDA designated teprotumumab as a “breakthrough” therapy for TAO based on the findings of that trial.

### 5.2. Phase 3 Trial

A follow-up phase 3 trial was organized following the publication of the initial trial and the transfer of teprotumumab to a new sponsor. The study was funded by Horizon Pharmaceutical (currently Horizon Therapeutics). It was designed similarly to that of the earlier study and was multicenter across North America and Europe. It involved a subset of performance sites conducting the phase 2 trial [101]. Enrolment began October 24, 2017 and ended August 31, 2018. A total of 107 patients were screened and 83 underwent randomization. The inclusion and exclusion criteria were similar to the Phase 2 trial but individuals with history of inflammatory bowel syndrome were excluded. The trial included an extension trial for patients failing to respond at week 24, regardless of whether they received a placebo or active drug. Thus, all proptosis non-responders were eligible for participation in this open-label extension study (OPTIC-X (NCT03461211). The extension included eight additional infusions of teprotumumab administered over 24 weeks. Randomization of phase 3 was stratified to equally distribute smokers to the two treatment arms. The primary endpoint of the study was modified to the proptosis responder rate that is the percentage of patients with ≥2 mm proptosis reduction in the study eye without ≥2 mm increase in the fellow eye at week 24 comparing those receiving teprotumumab versus placebo. Among the secondary outcomes was (1) the overall responder rate, which was the primary outcome in the phase 2 study. It is defined as percent of patients exhibiting both ≥2-point reduction in CAS AND ≥2 mm reduction in proptosis in the study eye. This must occur in the absence of a corresponding worsening in the fellow eye; (2) percentage of patients achieving CAS 0 or 1; (3) diplopia improvement at week 24 of at least 1 point on the Gorman scale; (4) change in GO-QOL overall score through week 24. Participants not included in the study extension were followed for 48 weeks. Those relapsing during this follow-up after with responded initially were also offered participation in the extension study.

**Figure 6:**
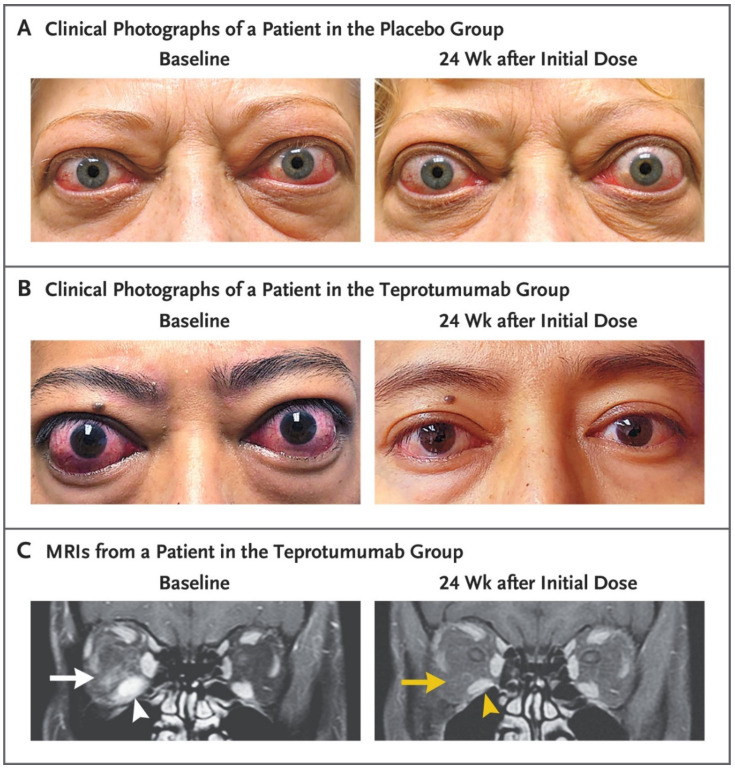
Photographs and MRIs at Baseline and 24 Weeks after receiving either placebo or teprotumumab in the Phase 3 trial. (**A**) Facial images of a patient receiving placebo. At baseline, substantial proptosis (left eye, 29 mm and right eye, 27 mm) and inflammatory signs (left eye Clinical Activity Score of 7 and right eye 5) are present. At week 24, proptosis and inflammation remain. (**B**) Facial images of a patient receiving teprotumumab. At baseline, proptosis (both eyes 24 mm), edema, upper and lower eyelid retraction, and multiple inflammatory signs (CAS 5 bilaterally) are present. At week 24, bilateral reductions in proptosis (−5 mm) and CAS (−4 points). (**C**). Contrast-enhanced, MRI shows fat-saturated T1-weighted coronal views in a single patient treated with teprotumumab at baseline and week 24. Enhancement of the inferior rectus muscle (white arrowhead) and orbital fat (white arrow) and nferior rectus muscle enlargement. At week 24, resolution of inferior rectus muscle (yellow arrowhead) enhancement and orbital fat (yellow arrow). Muscle volume was reduced by 49% (yellow arrowhead). Proptosis reduction decreased from 23 mm at baseline to 18 mm at week 24. From N. Engl. J. Med, Douglas R.S, Kahaly G.J., Patel A., Sile E.H.Z., Thompson R. et al. Teprotumumab for the treatment of active thyroid eye disease. 382; 341–352. Reprinted with permission from ref. [101]. Copyright 2020 Massachusetts Medical Society.

9.5% of patients in the placebo cohort responded at week 24 while 82.9% of those receiving teprotumumab achieved the primary response (delta 73.45%; 95% CI 58.89% to 88.01%; *p* < 0.001). The majority of patients responding at week 24 had already responded at week 6. Mean proptosis reduction among those receiving active drug was −3.32 mm at week 24 or a mean difference from placebo of 2.79 mm. Achievement of all secondary outcomes was significantly greater among those receiving teprotumumab. That treatment group achieved CAS scores of 0 or 1 more frequently as well as achieving over-all responses. Diplopia improvement of ≥1 Gorman grade occurred in 50.0% of the 28 patients with baseline diplopia in the teprotumumab group by week 6 compared to 3.6% of the 28 diplopic patients receiving placebo. Off-protocol orbital imaging was performed at baseline and again at week 24 of six patients (Figure 6A–C). Decreased extraocular muscle volume, primarily of the inferior rectus muscle was detected in 4 of 6 patients. Reduction in orbital fat volume and stranding was also detected in two patients. It would thus appear that teprotumumab treatment results in volume reduction of both extraocular muscles and orbital fat in some patients with TAO.

## 6. Adverse Events

Several adverse events emerged from both trials. Most were mild to moderate and resolved spontaneously, either during or soon after the treatment phase was completed. Among these, hyperglycemia occurred, primarily among patients with either frank diabetes mellitus or carbohydrate intolerance diagnosed prior to trial participation. These cases were uniformly managed with adjustments in diabetes therapy if necessary, the medication requirements of which returned to baseline at the conclusion of treatment. There were no reported cases of ketoacidosis. Leg muscle cramps were not uncommon and did not require medical treatment. Hearing abnormalities were reported in some patients receiving teprotumumab, including tinnitus, hypoacusis, deafness, and autophony. Audiology investigations failed to reveal their etiology. These cases also resolved spontaneously. Other adverse events included hair loss, diarrhea, and reactivation of inflammatory bowel disease in an individual with a history of ulcerative colitis.

## 7. The Future

Several real-world observations now suggest that teprotumumab may be effective in both compressive optic neuropathy [102,103] and chronic, stable TAO [104]. A very recent report suggests improvement in pretibial myxedema following treatment with the drug [105]. Thus, with regard to extremely aggressive TAO and that which has become clinically stable, teprotumumab may prove to be therapeutically beneficial. It is also possible that other diseases sharing a similar role of IGF-IR in its pathogenesis, such as rheumatoid arthritis [106] may also prove responsive to the inhibition of the receptor. Even beyond the scope of diseases typically considered to be autoimmune, potential therapeutic responses might be anticipated through the targeting of the IGF-I pathway and IGFBP3, a report by Lee et al. opined on the plausible treatment of asthma with these and related approaches [107], given the pathway’s putative role in mediating airway hyper- responsiveness, fibrosis and inflammation.

## Data Availability

Not applicable.

## Abbreviations

EGF	Epidermal Growth Factor
EGFR	Epidermal Growth Factor Receptor
ERK	Extracellular Signal-Regulated Kinase
GH	Growth Hormone
GPCR	G-protein coupled receptor
IR	Insulin Receptor
IR-A	Insulin Receptor A
IR-B	Insulin Receptor B
IGF	Insulin-like Growth Factor
IGFBP	Insulin-like Growth Factor Binding Proteins
IGFBP-rp	IGFBP-related proteins
IGF-IR	Insulin-like growth factor-I receptor
IGF-IRβ	Insulin-like growth factor-I receptor beta subunit
IGF-II	Insulin-like growth factor-II receptor
Kd	Equilibrium dissociation constant
MAPK/MEK	Mitogen-Activated Protein/Extracellular Kinase
MTor	Mammalian Target of rapamycin
PI3K	Phosphatidylnositol-3- Kinase
Raf	Rapidly Accelerated Fibrosarcoma
Ras	Rat Sarcoma
RTK	Receptor Tyrosine Kinase
TAO	Thyroid-associated ophthalmopathy
TCF	T-Cell Factor
TSH	Thyroid-Stimulating Hormone
TSHR	Thyroid-Stimulating Hormone Receptor
